# What the study of spinal cord injured patients can tell us about the significance of the body in cognition

**DOI:** 10.3758/s13423-022-02129-6

**Published:** 2022-06-13

**Authors:** V. Moro, M. Scandola, S. M. Aglioti

**Affiliations:** 1 Department of Human Sciences, NPSY.Lab-VR, University of Verona, Lungadige Porta Vittoria, 17, 37129 Verona, Italy; 2 IRCCS Santa Lucia Foundation, Rome, Italy; 3 Department of Psychology, University “La Sapienza”, Roma and Istituto Italiano di Tecnologia, Rome, Italy

**Keywords:** Embodied cognition theories, Spinal cord injury, Deafferentation and deefferentation, Body in the mind

## Abstract

Although in the last three decades philosophers, psychologists and neuroscientists have produced numerous studies on human cognition, the debate concerning its nature is still heated and current views on the subject are somewhat antithetical. On the one hand, there are those who adhere to a view implying ‘disembodiment’ which suggests that cognition is based entirely on symbolic processes. On the other hand, a family of theories referred to as the Embodied Cognition Theories (ECT) postulate that creating and maintaining cognition is linked with varying degrees of inherence to somatosensory and motor representations. Spinal cord injury induces a massive body-brain disconnection with the loss of sensory and motor bodily functions below the lesion level but without directly affecting the brain. Thus, SCI may represent an optimal model for testing the role of the body in cognition. In this review, we describe post-lesional cognitive modifications in relation to body, space and action representations and various instances of ECT. We discuss the interaction between body-grounded and symbolic processes in adulthood with relevant modifications after body-brain disconnection.

## (Dis)Embodied approaches to cognition

The traditional occidental concept of the human mind seems to be essentially based on mind-body dualism deriving from the Cartesian distinction between the mind (*res cogitans*) and the body (*res extensa*). The mind-body dichotomy has been taken to imply not only that basic perceptual and motor functions are separated from higher order ones (Block, 1995), but also that the latter are exclusively based on the manipulation of abstract, amodal symbols and are largely independent from the former (Newell & Simon, 1972). In the last few decades, this radical view has been challenged by ever increasing psychological and neuroscientific evidence that human cognition is profoundly influenced by basic sensorimotor processes and that even complex concepts such as the abstract aspects of language are largely grounded on body representations and their relations with the world. This is the central tenet of a group of theories that are included under the umbrella definition of ‘Embodied Cognition Theories’ (ECTs). According to these theories, all human experience is grounded in the body, not only perceptual and emotional processes and social interactions, but also the acquisition and creative use of language (e.g., the use of metaphors), judgment capacities and the creation of cultural artefacts (Gallagher, 2005). Since their original formulation (Glenberg, 1997), ECTs have attracted the interest of many disciplines, such as psychology, psychotherapy (Khoury et al., 2017; Tschacher et al., 2017), education (Pouw et al., 2014), philosophy, anthropology, robotics (Hoffmann et al., 2010), artificial intelligence (Shapiro, 2011) and, last but not least, neuroscience (Freund et al., 2016; Kiefer & Pulvermüller, 2012; Mahon & Caramazza, 2008). However, ECTs do not refer to a unitary construct and each theory does in effect differ from another in the way it conceives the reciprocal relations between the body, the mind and the environment and the modalities by means of which bodily representations affect cognition. The various different theories range from a general idea of an instrumental role of the body in information processing (Körner et al., 2015) to a more radical view asserting that “all cognitive processes are based on sensory, motor and emotional processes, which are themselves grounded in body morphology and physiology” (Glenberg, 2015, p. 166).

**Fig. 1:**
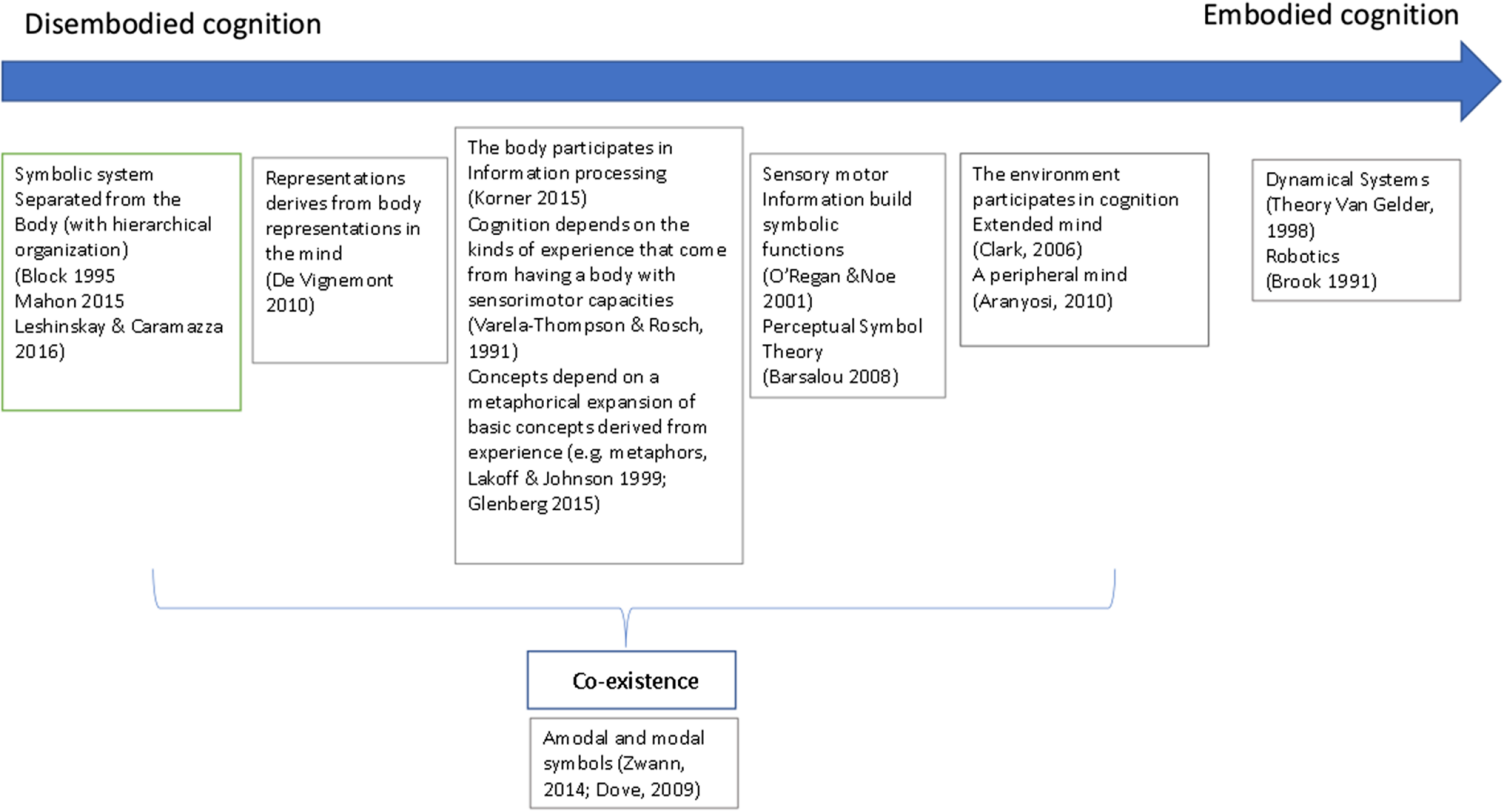
The various different models of embodied cognition theories are represented in a progression from one extreme with Disembodied Cognition to the other extreme positions inside the Embodied Cognition Theories. The co-existence of modal and amodal symbols in adulthood is suggested

Importantly, however, a sort of continuum is identifiable within these various theories (Fig. 1). At one extreme of this continuum, there is a hypothesis that presupposes the hierarchical organisation of cognition with a symbolic system that is separated from the sensorimotor system that can merely activate motor responses (Leshinskaya & Caramazza, 2016). At the other extreme is the idea that cognition emerges from a dynamic circle of interactions between the brain, the body, and the environment without the need for symbols (Brooks, 1991; van Gelder, 1998). What distinguishes these two perspectives regards the role that the body and its connection to objects plays in cognition (Shapiro, 2019). The body may be considered to ‘participate’ in building cognition since cognition may be altered depending on the shape, size and experiences of the body (Glenberg, 1997; Lakoff & Johnson, 1999; Varela et al., 1991). From a different perspective, the body can be considered to be ‘constitutive’ in the sense that cognition would not exist without it (e.g., the Perceptual Symbol theory; Barsalou, 2008; O’Regan & Noë, 2001). Objects are only taken into account in some of these theories in which it is suggested that they participate in building cognition (e.g., the Extended mind theory, Clark, 2006; the Dynamical systems theory, van Gelder, 1998). An example is the act of writing and thinking at the same time, a task that gives a specific result due to the interaction between the brain and the body and thence to a pen and paper, and from there back again to the brain (Clark, 2006). Accordingly, if one changes either the gesture or the object, the final product will also be different. One might ask whether in this case the mind extends to the body (e.g., the Peripheral mind theory, Aranyosi, 2013) and also to the objects (Clark, 2006) or, alternatively, the mind incorporates the body and the objects it is interacting with (Borghi, 2005). This is a question that remains unanswered.

Recent studies on the link between embodiment and higher order functions in people with sensory deprivation highlight the importance of both sensory and conceptual representations (Ostarek & Bottini, 2021). For example, anterior temporal lobe activation in colour-knowledge tasks turned out to be very similar in congenital and early blind subjects (Wang et al., 2020). In contrast, activation in the ventral occipito-temporal colour perception regions was found only in sighted controls. This pattern of results points to the existence of two forms of object representation in the human brain: a sensory-derived and a cognitive-derived form of knowledge (Wang et al., 2020), with the former being experience-dependent and the latter experience-independent (Ostarek & Bottini, 2021). Crucially, the analyses of connectivity in Wang et al.’s study shows that the two systems relating to colour knowledge are integrated and part of a widespread network (Wang et al., 2020). Thus, a crucial question concerns not only *whether* but also *how* the two levels interact and if the sensory level is able to modulate and modify the conceptual level. If so, one can conclude that knowledge is embodied, although embodiment is not the only way the brain understands the world.

While no single clinical condition makes it possible to distinguish between the various different ECTs, alterations in the body may provide novel information on the different variables that play a role in these processes. Studies of amputees, for example, may highlight possible representational bodily changes that might, however, be due to multiple aspects, such as the visual appreciation of conspicuous changes in body shape as well as the somatosensory and motor disconnection between the body and the brain. In the following section, we focus on individuals suffering from spinal cord injury (SCI) in whom the general body shape is unchanged in spite of a massive somatosensory de-afferentation and motor de-efferentation. The specificity of this neurological model with respect to other clinical conditions will be analysed, then the changes in cognitive functions associated with SCIs are reviewed, starting from the representation of static and acting bodies, and continuing with an exploration of object and space representations. The potential contribution of these experimental data to the debate on embodied cognition will conclude the review.

## Spinal cord injury (SCI) as a model for understanding the role of the body in cognition

**Fig. 2:**
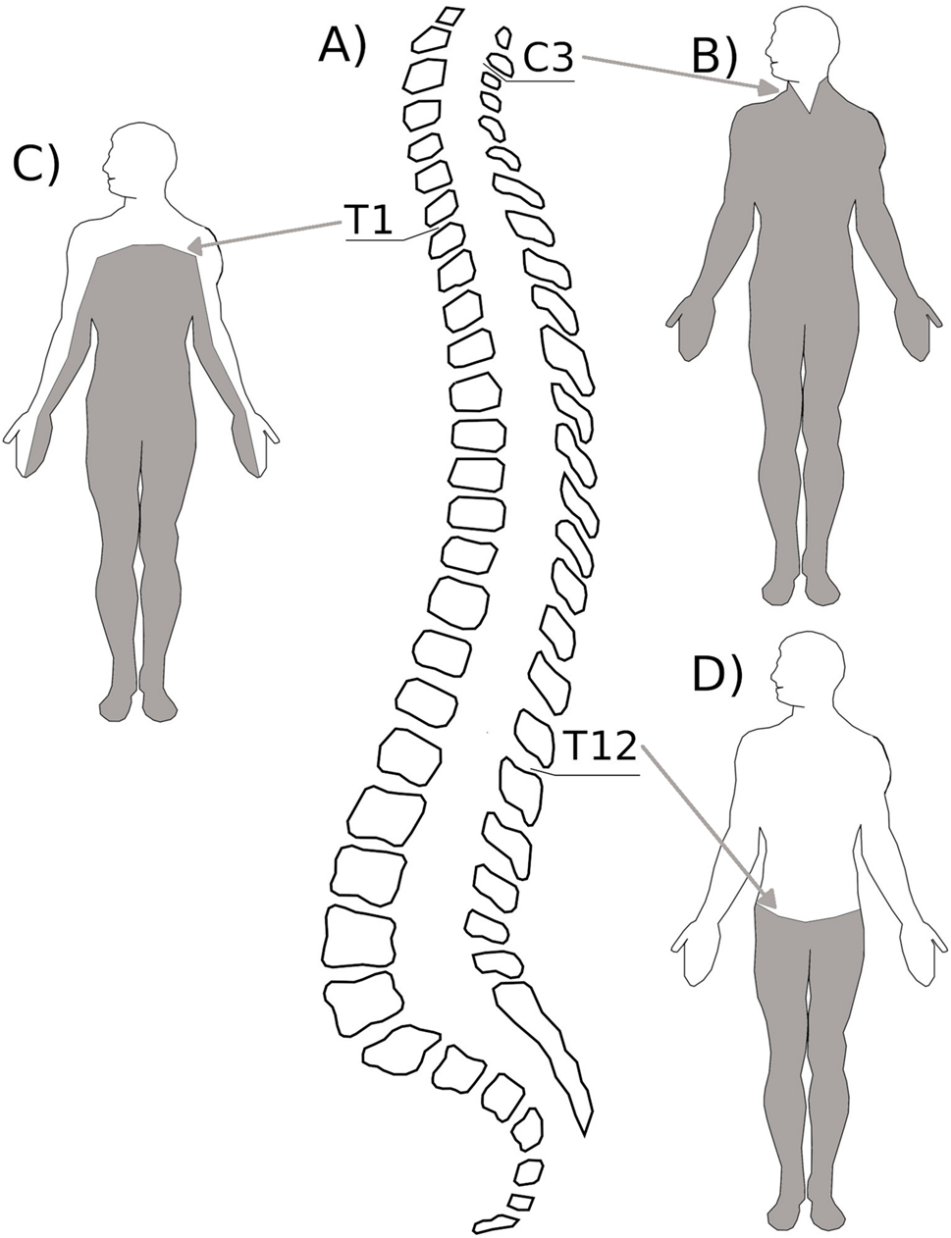
Graphical representation of the somatosensory and motor deficits following a SCI. **A**) The various levels of spinal cord are represented in the spinal column. The grey regions (**B**,**C**,**D**) indicate the body parts of altered processing of somatosensory and motor signals. **B** = cervical lesion with tetraplegia; **C** = thoracic lesion (T1) with paraplegia and partial deficit in the upper limbs; **D** = lower thoracic level (T12) with paraplegia

Spinal cord injury (SCI) is a clinical condition in which a complete or incomplete lesion of the spinal cord induces a total or partial interruption of the bidirectional communication between the body and the brain, with the consequence that no somatosensory input from the body periphery below the lesion level (e.g., sensations of touch, pressure, the sense of limb position) is sent to the brain (de-afferentation) and no motor commands from the motor cortices can reach the muscles controlling the body parts below-the-lesion level, ultimately leading to paresis or paralysis (de-efferentation). The extent of deprivation depends on the lesion level. SCI at the cervical level brings about hypoesthesia/anaesthesia and tetraparesis/tetraplegia, a clinical condition with impaired/absent somatosensory and motor functions affecting both upper and lower limbs (Fig. 2). SCI below the first thoracic spinal cord segment leads to paraparesis/paraplegia (i.e., the deficits affect lower limbs but spare upper limbs, neck and head regions). Given the topographic organisation of the spinal cord, exploring patients with lesions at different levels makes it possible to investigate in the same individual the representations of the body parts that are de-afferented/de-efferented and those that are still connected to the brain. For example, in patients with high cervical lesions, the face and head regions are normally connected to the brain while the body regions below the neck are disconnected from the brain; in patients with lesions affecting the lumbar region, the deprivation exclusively involves the lower parts of the body. It is exactly this topography of damage that offers a unique opportunity to investigate the specificity of deprivation-related changes in cognition.

Furthermore, a second aspect that makes the SCI model interesting and contributes towards a better understanding of the relevance of body in cognition is that the lesion is confined to the spinal cord and does not affect the brain. In this way, any cognitive changes recorded after lesion onset cannot be attributable to brain damage but are instead clearly the indirect effect of somatosensory and motor disconnections. To date, the link between the body and cognition has been investigated in patients suffering from brain damage (e.g., Canzano et al., 2014; Cocchini et al., 2010; D’Imperio et al., 2017; Fossataro et al., 2018; Garbarini et al., 2015; Moro et al., 2011; Moro, Urgesi, et al., 2008b; Pazzaglia, Pizzamiglio, et al., 2008a; Tosi et al., 2018). However, the mere presence of brain damage makes it difficult to understand the potential specific role of body afferences and efferences in modulating cognitive functions. In fact, when analysing the results from tests carried out with brain injured participants, three limitations need to be taken into account. Firstly, it is always necessary to consider the extreme variability between patients with regard to their symptoms, even when they share the same diagnosis. Secondly, it may be difficult to distinguish the symptoms that directly depend on the lesion and the changes that are secondary to the somatosensory-motor disability. Thirdly, brain lesions may affect neural networks that extend beyond specific cortical regions and involve the white matter tracts and thus the connections between distant, not directly damaged brain areas. This induces alterations which cannot be explained in terms of mere lesion site but rather in terms of a disconnection between cortical regions, even those which are remote from one another (Pacella et al., 2019, 2020; Thiebaut de Schotten et al., 2015). These limitations are overcome in cases of SCI in whom there is no primary brain damage.

The SCI model also has advantages over other clinical conditions which involve body de-afferentation and de-efferentation without direct, primary brain damage, but with clear signs of plastic remapping of sensorimotor and cognitive functions. For example, evidence of fast, profound somato-topography in remapping processes regarding body and space representations has been discussed in studies on individuals who have undergone amputation (Aglioti et al., 1994; Aglioti et al., 1997; Canzoneri et al., 2013; Ramachandran et al., 1992). It is worth noting that, unlike spinal cord lesions, amputations involve the real loss of a body part and, as a consequence, a conspicuous change in the shape of the body. Thus, any changes in cognition observed in amputees may at least in part depend on other non-somatosensory and motor factors, such as, for example, vision-mediated body representations. Conversely, in spinal lesions, the body shape is not altered, and any effects found in cognitive functions only depend on somatosensory-motor disconnection.

A condition of massive deprivation that may permit an exploration of how alterations in the link between central and peripheral systems modulate cognition is *amyotrophic lateral sclerosis*, a neurodegenerative condition characterised by selective damage to motor neurons. However, this pathological condition induces a selective de-efferentation as a consequence of degeneration of the motor neurons connecting the motor cortices and the spinal cord, as well as those connecting the spinal cord to the muscles. Importantly, the degenerative nature of the pathology makes it difficult to classify the cognitive changes which frequently occur, in particular with regard to executive functions (Gillingham et al., 2017) and language (Abrahams et al., 2014), as symptoms resulting directly from the disease or as a consequence of motor deficits.

A form of body-brain disconnection that may turn out to be even more massive than that which can be seen in SCI regards patients with locked-in syndrome in which changes in body representation have been documented (Conson et al., 2008; Conson et al., 2010; Pistoia et al., 2010). However, patients with locked-in syndrome typically have brain-stem lesions with the possible involvement of complex central hodological pathways. Moreover, an in-depth investigation of cognitive changes in these patients is difficult due to the rarity of the syndrome and the problems of establishing appropriate communication modalities in the experimental context (Rousseaux et al., 2009; Schnakers et al., 2008).

On the whole, these considerations indicate that SCI may be considered as a unique model for testing the strengths and limits of embodied approaches to cognition. In fact, if cognition is in some way grounded in the link between the brain, the body and the environment, changes in the somatosensory-motor capacity to act and perceive the world should also lead to changes in cognitive functions. Moreover, since the disconnection in SCI is topographically organised (i.e., it typically involves some body parts and not others), one may observe, at least in principle, that changes in cognitive functions may be contingent on functions associated with the disconnected body parts. This hypothesis has been explored in studies that investigate the visual discrimination of body part, space and action representations as well as motor imagery. These will be discussed in the following sections with reference to theories regarding embodied cognition and its implications for further research.

## Sensorimotor pathways to mental body representations

Before discussing the role of the body in cognition, it may be useful to analyse the ways in which the SCI model can give us information about how the body is represented in the brain (Berlucchi & Aglioti, 2010). The relationship between the body and the brain is a difficult matter, as a basic ambiguity persists that seems to be hard to resolve: the brain is an organ of the body, but at the same time it is capable of representing the body (Berlucchi & Aglioti, 2010; Tsakiris & Haggard, 2010). As a result of this complexity, an important question becomes how top-down (from the brain to the body) and bottom up (from the body to the brain) processes interact in the building of body representations and how this balance may be altered in SCI.

According to the Perceptual Symbol Theory (Barsalou, 2008), cognitive symbols are actually simulations of sensorimotor and inner (e.g., interoceptive) states, thus suggesting that even seemingly ineffable constructs are built and maintained by means of bodily signals. In fact, experiences create ‘sensorimotor contingencies’, that is, a set of rules and regulations that relate sensory inputs to movements, postural and interoceptive changes and actions. Sensorimotor contingencies are independent from consciousness but crucially impact on it; in fact, with a contribution from inner states (i.e., emotions, memories, etc.), sensorimotor contingencies build perceptual states, which may be then simulated by the brain during cognitive activity (Barsalou, 2008).

An apparent independence of sensorimotor contingencies from cognition may be found in procedural learning, namely, the implicit ability to learn motor sequences (e.g., driving a car or playing a musical instrument). In these processes, the contribution of an ‘explicit’, verbal cognition is very limited, if not downright confusing. Thus, procedural learning suggests that “what individuals are doing” is in some way separate from their knowledge of “how they do it”. In other words, unlike declarative learning, procedural learning does not need any cognitive symbols or any manipulation of these symbols, suggesting that somewhat complex cognitive operations can be performed thanks to bodily signals.

This independence of the body from cognitive representations is however a matter of debate. De Vignemont, for example, asserts that cognition “can be said to be embodied because it is affected not directly by the body but by the way the body is represented in the mind” (de Vignemont, 2011, p. 4), as in experiences of disownership of one’s own limb (Jenkinson et al., 2018; Moro et al., 2016). In this condition, “there is not only the absence of the experience of ownership but also the experience of its absence” (de Vignemont, 2011, p. 23). This sensation of absence, according to De Vignemont, is mediated by the cognitive representations of the body. From this perspective, a stream of consciousness from the brain to the body seems to characterise the experience of the body (or its deficits) based on its representations.

However, studies on SCI indicate that another parallel stream flows from the body to the brain and that sensorimotor peripheral changes can modify the cognitive representations of the body.

Indeed, some studies suggest that afferences and efferences play a pivotal role in building and maintaining these representations of the body (Facchin et al., 2021). In the following two paragraphs, these aspects are discussed. In section 3.1, we present evidence regarding the effects of the lack of bottom-up sensory information in the multisensory visuo-tactile integration of information coming from the body (assessed by means of the rubber hand illusion). In section 3.2, we show how these effects expand beyond the sensorimotor domains toward cognitive functions such as complex body-related visual discriminations, indicating that sensorimotor variables modulate higher-order representations**.**

### The rubber hand illusion

People with SCI report experiences of corporeal illusions, in particular distortions related to their body (Conomy, 1973; Curt et al., 2011; Scandola, Aglioti, Avesani, et al., 2017a), such as for example, *disownership*-like feelings (i.e., the feeling that some body parts do not belong to the self) and *somatoparaphrenia*-like sensations (e.g., the occurrence of delusional ideas relating to body part misidentification, personification or objectivation). Unlike brain injured patients, in whom these symptoms have long been described as a consequence of specific cerebral network alterations (for a review, see Jenkinson et al., 2018; Romano & Maravita, 2019; see also Moro et al., 2016) and are associated with delusional beliefs (i.e., convictions that are not amenable to change despite conflicting evidence), people with SCI recognise the irrationality of these body-generated feelings, which, however, they are unable to control.

In apparent contrast with these corporeal illusions that are activated by a body-brain disconnection, it is worth noting that SCI individuals are less sensitive than healthy people to bodily illusions when these are induced by external stimuli (e.g., tendon-vibration; Fusco et al., 2016). In fact, when engaged in experimental paradigms, SCI patients respond to questions regarding their body basing their reply on cognitive representation and semantic information rather than on information coming from their body. For example, when they are asked to estimate the spatial position of a body part that is hidden from view (e.g., their hand), they base their judgment on their cognitive knowledge of the usual position of that body part, and do not change their estimation after spatial manipulation or a drift of the very same body part (Scandola et al., 2014). It seems as if the balance between body functions and their representation is lost, with the prevalence of a given component in depending on the situation.

The result is a type of imbalance between peripheral inputs and central representations that has been explored by means of the *Rubber Hand Illusion* (RHI). In the RHI paradigm, a visuo-tactile conflict is induced leading to rapid changes in the sense of body ownership (Botvinick & Cohen, 1998). In RHI studies, healthy individuals were asked to look at a rubber hand that was being stroked by an examiner, synchronously or asynchronously with their own hand, which was hidden from view. It turned out that only during synchronous stimulation was the rubber hand perceived as part of the participants’ own body (i.e., a subjective index of ownership over an artificial hand) and the position of the real hand was perceived as having shifted toward the rubber hand (‘proprioceptive drift’, an objective index of illusory perception of body in space; Botvinick & Cohen, 1998; Ehrsson et al., 2005; Longo et al., 2008; Mohan et al., 2012; Schaefer et al., 2013; Tsakiris & Haggard, 2005). SCI participants with incomplete lesions tested in a RHI paradigm showed subjective indices of illusory hand ownership comparable to healthy participants (Lenggenhager et al., 2012). However, when the illusion was induced on their de-afferented legs, the SCI patients were less sensitive than the controls to multisensory stimulations (leg-illusion; Pozeg et al., 2017, see section 4.1). This supports the role of peripheral sensorimotor information in maintaining body representations.

Studies of the RHI in SCI patients are in line with reports of the plastic remapping of bodily representations in limb or finger amputees. In these studies, tactile stimuli delivered to the face ipsilaterally to the amputation side brought about the sensation of being touched not only on the face but also on the phantom hand (Ramachandran et al., 1992) or finger (Aglioti et al., 1997). Given the representational contiguity of the face and the hand in the cortical somatosensory system, the results have been interpreted as supporting the existence of perceptual correlates of post-ontogenetic plasticity. In a similar vein, a vertical version of the RHI paradigm has been used in paraplegic and tetraplegic patients in whom synchronous and asynchronous stimuli are administered to a fake hand and to the subjects’ ipsilateral hand or face. The results indicate that the tetraplegic (but not the paraplegic) participants reported the RHI when their ipsilateral face was stimulated. That only the patients who had lost the functionality of their hands were prone to the illusion suggest that the de-afferented hand region was willing to be driven by facial input with the consequence of across body region remapping processes (Scandola et al., 2014; Tidoni et al., 2014).

Taken as a whole, these data demonstrate that the interruption of body-brain connections induces not only below lesion, sensory and motor deficits as expected but also changes in the brain networks involved in body representations, supporting the embodied cognition approach. Tellingly, these changes expand beyond the multisensory integration (as assessed in the presence of multisensory illusions) which involves higher-order processes, such as complex body-related visual discrimination, which will be discussed in the next section.

### Visual discrimination of the human body

**Fig. 3:**
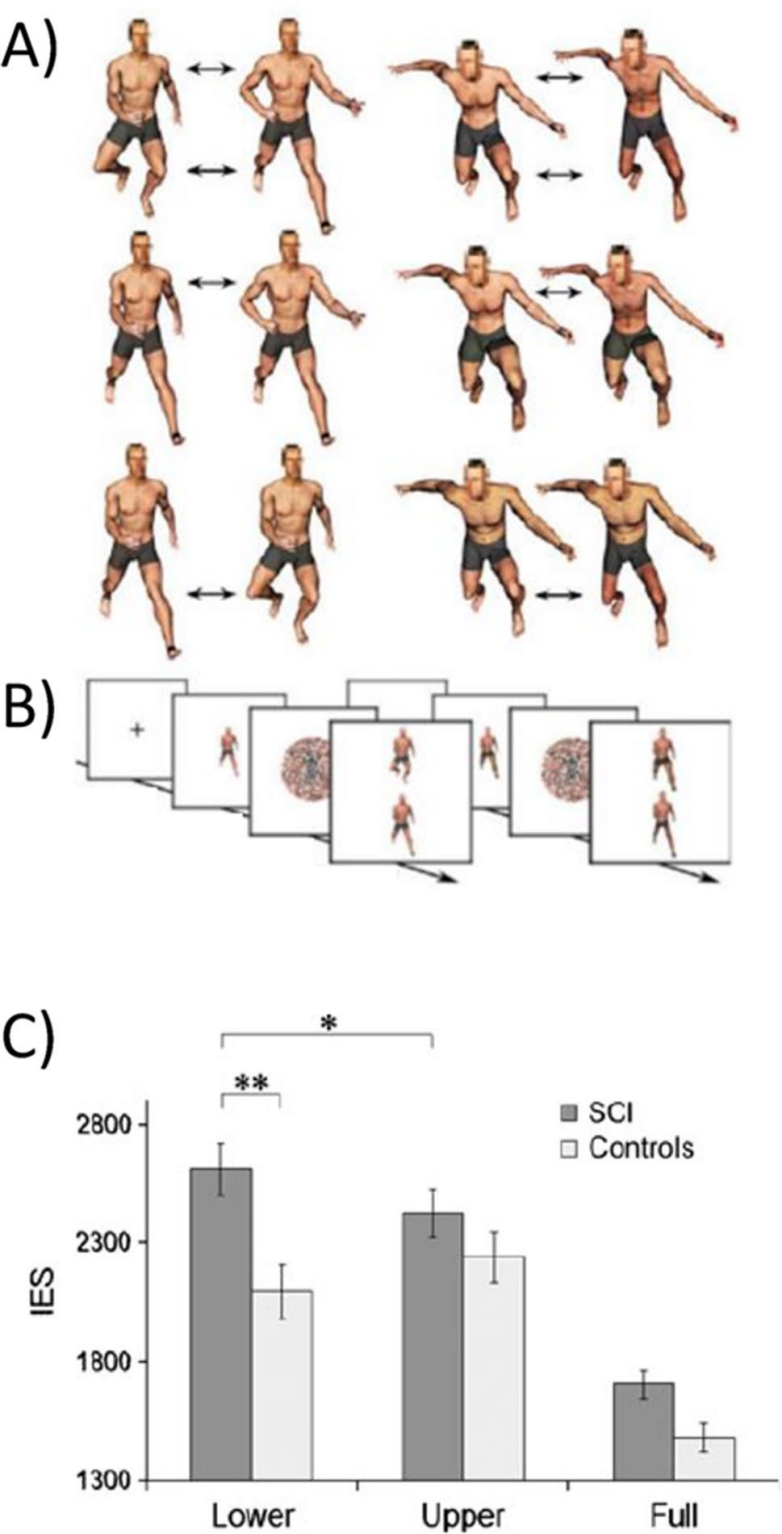
Changes in the visual discrimination of body form and action (modified from Pernigo et al., 2012). **A** = graphical representation of the stimuli used in the study. In the two columns on the left, changes in actions are shown (upper line = changes in both the upper and lower body parts; in the middle = changes in the upper body parts; lower line = changes in the lower body parts). In the two columns on the right the images differ in the form (i.e., identity) of the model but not in their action. **B** = experimental timeline. **C** = main results indicating a reduction in body representation of lower limbs in spinal cord injury

It is worth noting that until very recently, alterations in body representations were only discussed in relation to patients affected by brain damage and they were interpreted as being the result of modifications in specific networks (Jenkinson et al., 2018; Romano & Maravita, 2019). Interestingly, SCI studies also hint at a topographical remapping of non-de-afferented/de-afferented body parts in domains other than somatosensory perception and motor control. Pernigo et al. (2012), for example, asked people with SCI to discriminate visual stimuli representing human bodies that differed both in shape and in the action they were performing. In a matching-to-sample task, the paraplegic participants looked at an image of a young man performing a movement and were asked to choose the identical image from among two images that were presented immediately after the first (Fig. 3). The differences between the two images were in the upper or lower body parts and regarded the body form (i.e., the choice was between two individuals performing an identical action) or action (i.e., the choice was between two images of the same individual performing two different actions). The SCI participants performed worse than the healthy controls only when they were requested to identify differences in the lower body parts (which in fact corresponded to the paralysed parts of their own body). In contrast, the patients’ performance was similar to that of the controls for healthy upper body parts (Pernigo et al., 2012). The same trend was found in tasks involving the discrimination of body form and action (see below for a discussion regarding action perception; Fig. 3). It is worth noting that this was a visual discrimination task and thus apparently did not involve the sensorimotor system. Interestingly, the only way to understand these results is to posit that a sensorimotor simulation is activated during the task and thus that the body sensory-motor system participates in a cognitive function such as visual discrimination.

A deterioration in whole-body representation in people suffering from SCI was also found in a study in which participants were asked to judge the laterality of rotated images of feet, hands and whole bodies while they were in two different postures (with their hands and feet held either (i) straight or (ii) crossed). A posture-dependent modulation in reaction times in terms of the mental rotation of the body parts in the images was interpreted as the effect of afferent somatosensory information relating to body representation. In fact, this result was found for the control group, but not for the paraplegic group for whom the effects of postural feet changes disappeared and the body representation progressively deteriorated in proportion to the degree of completeness of their SCI (Ionta et al., 2016; Scandola, Dodoni, et al., 2019b). These data confirm evidence found in a study involving a similar task carried out by patients suffering from *focal hand dystonia* (i.e., a group of movement disorders characterised by sustained or intermittent muscle contractions causing abnormal, often repetitive, movements, postures, or both; Albanese et al., 2013). Their responses were only slower when the body parts that they were requested to rotate corresponded to their own affected body parts (i.e., the dystonic hand), but not to their other hand or foot (Fiorio et al., 2006).

As a whole, these results suggest that changes in the body-brain relationship impact not only somatosensory and motor processes but also higher-order functions. A theoretical explanation for these results is offered by the Perceptual Symbols Theory (PST; Barsalou, 2008), according to which all cognitive experiences are necessarily grounded in the sensory and motor contexts of their occurrence. During the experience, sensorimotor codes are recorded as multimodal perceptual, motor and interoceptive states. These codes shape conceptual representations. When similar representations are reactivated on other occasions, these are based on a new access to the sensorimotor information previously encoded (motor simulation). Thus, concepts are not an additional level of abstract, amodal representation that is separated from sensorimotor systems, but they use the same neural and cognitive processes used in sensorimotor processing (Barsalou, 2008; Barsalou, 2010). People with SCI are unable to simulate the states of the below-the-lesion body parts that are deprived of bodily outputs and somatosensory inputs. As a consequence of this deprivation, they fail in tasks requiring representations of those parts. Tellingly, the fact that changing sensory-motor potentialities alters cognition does not fit in with amodal theories of cognition. In fact, following an amodal approach, once built, symbolic representations should remain stable and not change due to ‘peripheral’ body changes. A representation of the body that is exclusively symbolic is not expected to be sensitive to sensorimotor influences, as happens in the case of SCI.

## The body in the world of people and objects

When considering the role of the body in cognition, one needs to take into account that the body acts and perceives in specific contexts. According to the embodied cognition approach, this means that information from the environment contributes towards shaping cognition by means of the mediation of the body (Clark, 1999). The first consequence of a relationship between the kind of body that an organism possesses and the kind of concepts that the organism can acquire is that “to conceive of the world as a human being does require having a body like a human being’s” (Shapiro, 2011, p. 71). According to this view, the organism’s understanding of the world and its ways of categorising experiences are determined by the properties of the body. From this perspective, even in the case of language, which might be considered to be the most amodal, symbolic human function, the basic concepts would derive from physical experiences (e.g., Lakoff & Johnson, 1980; Liuzza et al., 2011).

In the following section, we discuss experimental results which indicate how changing sensorimotor bodily functions may alter the perception of the environment and objects. Two embodiment processes are analysed: the embodiment of artificial virtual agents (avatar) and the embodiment of objects that may or may not be in contact with the body and may or may not subserve adaptive navigational or motor functions. Furthermore, the effects of object embodiment in the representation of space will be discussed.

### Embodying artificial agents

The investigation of experiences of ‘mediated embodiment’ (i.e., the technologically induced illusion of experiencing the body of an avatar as one’s own, independently of the technology used to produce the illusion; Aymerich-Franch, 2018; Aymerich-Franch & Ganesh, 2016) provides a relevant contribution to the comprehension of how embodiment mechanisms impact cognition. Studies indicate that artificial agents, be they digital (i.e., an avatar) or physical (i.e., a robot), can be embodied to varying degrees. The sensations produced by this process are so strong that they may influence cognitive and social behaviour (Cangelosi & Stramandinoli, 2018; Wykowska et al., 2016). For example, embodying adults in the body of a 4-year-old child (Banakou et al., 2013) or a small mannequin (a ‘Barbie doll’; van der Hoort et al., 2011) causes an overestimation of object sizes. In contrast, object size is underestimated when adults are embodied in a giant body (van der Hoort et al., 2011). Furthermore, white people embodied in a black virtual body (Banakou et al., 2013; Peck et al., 2013) or ‘enfacing’ a black face (Bufalari et al., 2014) exhibit a decrease in implicit racial bias.

Embodiment of an avatar has been induced in patients with SCI by means of the Virtual Leg Illusion (Pozeg et al., 2017). Synchronous and asynchronous visuo-tactile stimulation was applied to the participants’ back, above the lesion level or at the shoulder (where afferences are spared, in the case of cervical lesions), while the virtual legs were seen on a head-mounted virtual-reality (VR) display in order to induce a visuo-tactile integration. People with SCI were less sensitive to the multisensory stimulations used to induce illusory ownership of the virtual legs than the controls, thus hinting at a specific role of body afferences and efferences in the illusion. Furthermore, the virtual-leg-illusion (as well as the full-body illusion, in which people look at the full body and not only the legs) was associated with a mild effect of pain reduction (e.g., Pozeg et al., 2017). This effect was confirmed in patients suffering from complex regional pain syndrome and peripheral nerve injury (Matamala-Gomez et al., 2019).

An in-depth knowledge of the processes involved in embodiment may have an enormous impact on future clinical practices, in particular on the possibility of creating brain computer interfaces and robotic tools that can be useful in rehabilitation after SCI (Jarosiewicz et al., 2015; Osuagwu et al., 2016; Sørensen & Månum, 2019). In particular, a more profound knowledge of the mechanisms involved in embodiment might help clinicians to understand why some patients, for example amputees, express negative attributions towards their prosthesis (Senra et al., 2012) and may suggest a type of therapy that will facilitate acceptance (Holthe et al., 2018). The implications of these procedures in terms of cognition are to date unknown and future research is needed (Gorgey, 2018; Lee et al., 2019). Two complementary areas of interest emerge from the literature on embodiment processes. The first regards the need to take into account data concerning the changes in body-related cognitive functions as a result of de-afferentation and de-efferentation in order to design optimal robotic devices. The second concerns the necessity to investigate the effects of the use of robotic aids on cognitive functions (Beckerle et al., 2019).

### The embodiment of objects

Clinical studies indicate that objects may be incorporated. Aglioti et al. (1996), for example, reported on a woman with right brain damage affected by disownership of her left hand who also denied the ownership of the rings she wore on that hand. When the same objects were put on her right hand or were held by the examiner, the patient correctly recognised them as her own. Other personal objects unrelated to her left hand (e.g., pins, earrings, a comb) were always correctly recognised as being hers. This indicates that the mental representation of one's own body may include inanimate objects that have been in contact with or in close proximity to the body itself. This has been confirmed in subsequent studies on body representation and peripersonal space (PPS; i.e., the region of space within which objects can be grasped and manipulated without the need to move the trunk). For example, the extensive use of a computer mouse extends the peripersonal space around the hand and this enlargement may include the screen monitor, at least during the time when the hand remains in contact with the mouse (Bassolino et al., 2010). Furthermore, in the case of blind people, the regular use of a cane to navigate extends their PPS to the full length of the cane (Serino et al., 2007), and the same occurs in amputees when they are wearing their prosthesis (Canzoneri et al., 2013). Similar plastic changes have been observed in healthy people after brief training sessions with a cane or two sticks, in particular when active movements with tools are requested (Maravita et al., 2002). In the same way, if a limb is forced into immobilisation, a reduction in the extent of the relative PPS occurs (Bassolino et al., 2014), along with a decrease in excitability at the cortical level (Facchini et al., 2002).

**Fig. 4:**
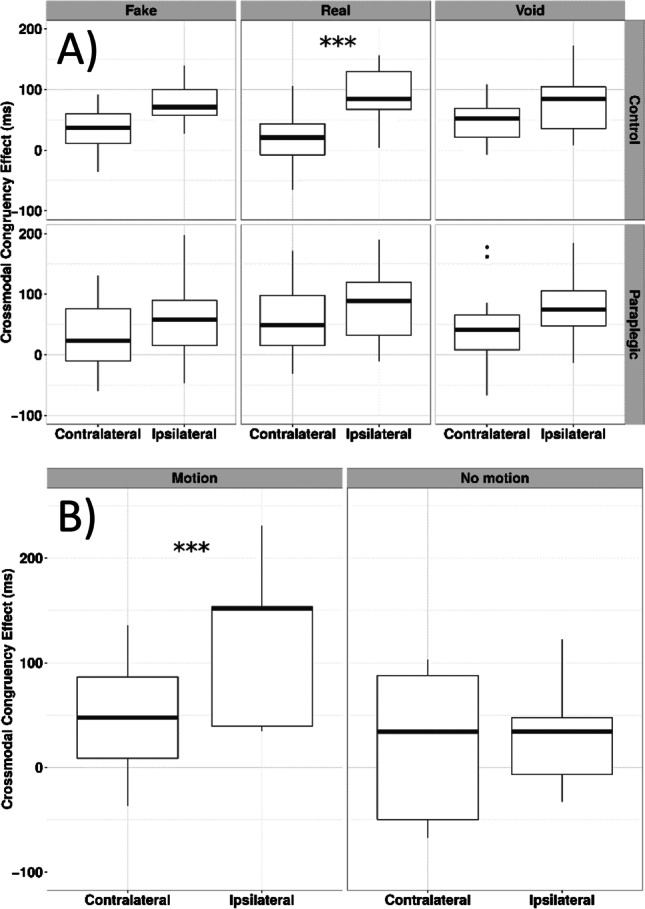
Changes in peripersonal space (PPS) around the feet (modified from Scandola et al., 2016). **A** = results in the comparison between spinal cord injury (SCI) and controls indicating a reduction of PPS around feet in SCI. **B** = PPS recovery of PPS representation around feet after mobilisation

Using a cross-modal integration paradigm in an experiment with paraplegic participants, Scandola et al. (2016) found that the somato-sensory and motor disconnection that characterises spinal cord lesions alters the representation of PPS in a specific way that impacts the space around the person’s legs that are paralysed (but not the space around the hands; Fig. 4).

In this case too, cognitive changes are not attributable to the generic adaptation processes that the individuals may have gone through in order to deal with their paralysis. On the contrary, the fact that these changes are somato-topographically specific suggests that sensory afferences and motor efferences do in fact play a causal role in building space representations and in rebuilding and adapting them to the changes which have occurred in the person’s body-environment relationship. In keeping with this view, there is evidence that 15 min of passive mobilisation are enough to bring about a recovery of the representation of space around the feet in paraplegics (Scandola et al., 2016). In healthy people, congruency in the visuo-motor information coming from the avatar in a VR setting seems to be necessary for embodiment and in fact the PPS turns out to shrink when this information is incongruent (Scandola et al., 2020). In contrast, in SCI patients, passive movement expands the PPS even in the absence of visual stimuli, indicating that the residual above-lesion sensory information probably plays a crucial role in PPS recovery after passive mobilisation (Scandola et al., 2020). It is thus evident that although top-down factors may partially modulate a patient’s response, they also certainly interact with information (or lack of information) coming from the body.

These studies support the theories suggesting that peripheral components involved in sensory experiences are not merely involved in the generation of experience, but are constitutive of experiences (O’Regan & Noë, 2001; Varela et al., 1991). A possibly extreme position is expressed in the Peripheral Mind Theory (Aranyosi, 2013), in which it is proposed that “there is a peripheral presence of the mind in every part of the body that gets innerved, and these peripheral parts of the mind are not less central than the processing center which is the brain” (page 11). According to this idea, the mind is multiply located or co-located. People feel that they are not in a body but are the body. Proprioception, interoception and touch all interact so as to create an “enminded body experience” (page 144). In this way, the mind extends beyond the brain to the body, in particular to the connections between the peripheral nervous system and the body.

The Extended Mind Theory (Clark & Chalmers, 1998) goes even further by claiming that cognition emerges from the interaction of individuals with the objects they use during cognitive activities. Clark suggests that gestures are a special reasoning system useful for spatial reasoning (Clark, 2008)*.* When a person writes what they are thinking at that moment, the paper provides a medium for thought thus enabling the person to shape and build their ideas. The same goes for tools that people frequently use as aids to cognitive functions, for example, the notebook that a patient suffering from Alzheimer’s disease uses in order to remember information plays the role usually played by memory networks (Clark, 2008).

So, the question now concerns whether it is the mind that extends out towards the world or the world that is embodied in the mind. The principle of ‘Economy of Action’ (Proffitt, 2006) has been used to study SCI in order to find an answer to this question. This principle considers perception to be embodied in an individual’s states, skills, goals and emotions. For example, the perception people have of the space surrounding them varies not only with any variation in the visual stimuli, but also with the individual’s intent to minimise the cost of their actions in that space in terms of energy – something that is mandatory for survival from an evolutionary point of view (i.e., Economy of Action). It has been shown that wearing a heavy backpack makes the perception of a distance farther (Proffitt et al., 2003) and the perception of an inclination of a slope steeper (Bhalla & Proffitt, 1999). The same happens when people feel fatigued due to the fact that they are maybe physically unfit or elderly or in bad health (Bhalla & Proffitt, 1999). In contrast, expertise in physical exercise or sport influences and ameliorates a person’s visual perception of any objects that have a key role in the sport they practise, for example, the hole in the course for golfers (Witt et al., 2008) or the ball for baseball players (Witt & Proffitt, 2005; but see Firestone, 2013, and Firestone & Scholl, 2016).

**Fig. 5:**
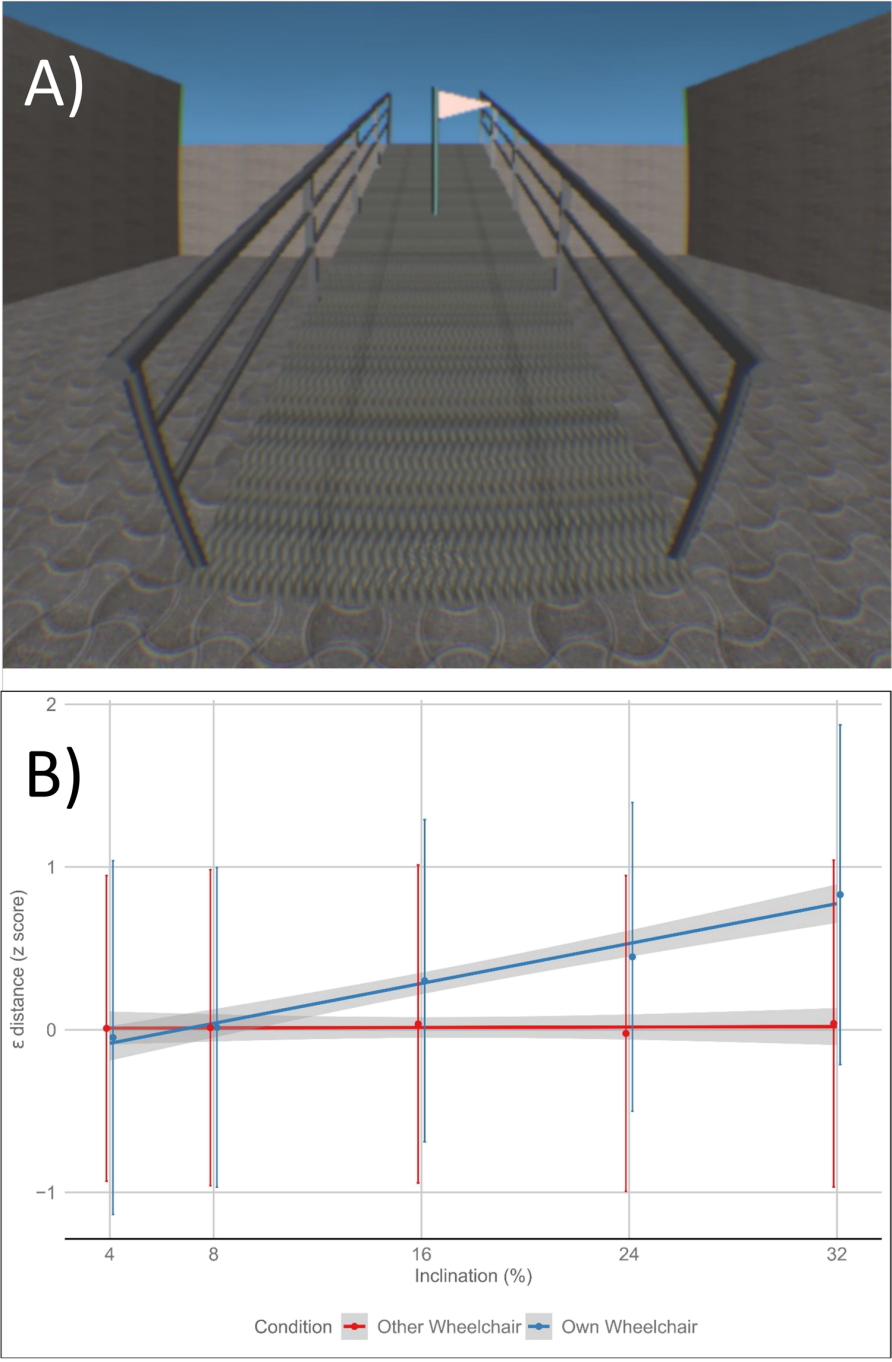
Changes in extrapersonal space representation in spinal cord injury (SCI) (modified from Scandola et al., 2019a, b, c). **A** = an example of the stimuli (a ramp with a flag) used in the experiment. **B** = the pattern of errors in perception of distances from the flag. As in healthy subjects, when SCI participants are seated in their own wheelchair, errors in estimation increase as the distance increases. This result suggests that coding the distance between a given point in space and own’s one position is based not only on visual estimation but also on body-related cues. It is worth noting that no such effect was found in SCI sitting in an unfamiliar wheelchair, suggesting that visual cues only are used in the absence of the association between one’s own body and an external object

In the case of people with SCI, an object of crucial importance for their autonomy in everyday life, and in general for their physical and social well-being, is the wheelchair. The interaction between the embodiment of a wheelchair and the Economy of Action principle was investigated with SCI patients (Scandola, Togni, et al., 2019c) to test the hypothesis that the greater the degree of embodiment of the wheelchair, the better the person would be at estimating distances in space. That is to say, if the body feels better due to embodiment, the person will be better able to judge distances. The Body View Enhancement Task was considered as a measure of wheelchair embodiment. In this task, the participants either sat in their own wheelchair or in one that they had never used before. They were asked to respond to flashing lights on their body parts both above and below the level of the lesion and on the wheelchair. Similar or slower reaction times (RTs) to stimuli on the body and the wheelchair indicated, respectively, the presence or absence of tool embodiment. In particular, if the RTs were similar between the body and the wheelchair, that would indicate embodiment. In contrast, if RTs were slower in the wheelchair compared to the body, that would indicate an absence of embodiment. The results indicated that the SCI participants embodied their own wheelchair but not the one they had never used before. Moreover, in keeping with Pozeg et al. (2017), the SCI participants displayed disownership of their lower limbs, which were treated as external objects (i.e., with responses slower than those given for the lights in the afferented body parts). Crucially for the aim of this review, embodiment of their wheelchair enabled the SCI participants to estimate physical distances in their extrapersonal space (as shown by means of a 3D virtual plastic scenario, see Fig. 5) as efficiently as the healthy controls (i.e., errors in estimation increased as the distance increased). This did not happen when they were in the unfamiliar wheelchair. In addition to demonstrating that the processes of tool embodiment impact on cognitive functions, the results support the hypothesis of a potential extension of body boundaries towards objects (i.e., embodiment) rather than an extension of the mind. In fact, the participants modulated their perception of space based on their actual body conditions in that moment, and wheelchair embodiment increased their perception of the space around them. According to Aranyosi (2013), “tools are more plausibly to be taken as part of the nervous system and hence the case is not of extended mind, but what I have dubbed ‘contracted world’, a case in which a previous autonomous part of the world gets ‘captured’ by a nervous system and so ceases to count as an external from then on“ (page 118). From this point of view, the mind is in effect extended but not beyond the body boundaries (or PNS).

Taken together, the results discussed in this section suggest that sensorimotor de-afferentation and de-efferentation extend their effects beyond one’s own body, in the representation of objects and environment. No cognitive changes at the level of the brain can explain these processes, which are totally mediated by the spinal cord lesion. The next step regards an investigation of potential changes in action representation and motor learning.

## How residual motor skills following SCI impact cognition

Monitoring one’s own performance is part of the interaction between the brain, the body and the environment. The ability to monitor skills is fundamentally important for regulating motor behaviour and learning. The complexity of the processes that underlie our daily-life decisions and actions in relation to objects and people may cause sub-optimal performance in a variety of circumstances leading to errors. Within the theoretical framework of the predictive brain (Friston, 2010), an error can be conceptualised as a deviation from one’s expectation based on previous (prior) knowledge about the regularity of events in the environment and in the social world. Detecting a mismatch between internal expectations (i.e., the underlying behavioural intention) and the actual spatio-temporal deployment of an event (e.g., perceived motor acts) is fundamentally important for updating and generating new internal models. Thus, performance monitoring has a crucial, adaptive role in forming, updating and using internal models concerning important aspects of behaviour (e.g., strong priors about what happens next).

Studying the development of children provides an important source of information for investigating the role of action in cognition. In fact, babies build some fundamental concepts and competences by means of their movements, actions and errors (Thelen & Smith, 1994), not only, for example, space and time representation, object and shape categorisation (Smith, 2005), abilities in mental rotation (Frick & Möhring, 2013) and the learning of foreign languages (Toumpaniari et al., 2015), but also in the development of scientific concepts (Kontra et al., 2015), decision making and choice selection (Rivière & David, 2013). At the opposite extreme, the cognitive deterioration and daily-life impairment associated with old age might be at least in part due to deficits in embodiment, which can in part be linked to neuronal degradation at the sensorimotor level (Kuehn et al., 2018).

Experimental studies also support the hypothesis of a causative role played by action in cognition (Schubert, 2004; Wells & Petty, 2010). Neurological patients demonstrate that the inability to perform actions also has consequences for higher-order non primarily motor tasks. Furthermore, patients affected by hemiplegia may show deficits in the visual discrimination of body parts, both in terms of action and form recognition (Moro, Berlucchi, et al., 2008a) and apraxic patients may be unable to recognise gestures (Canzano et al., 2014; Scandola et al., 2020, 2021; Zadikoff & Lang, 2005). Furthermore, an impairment in performing actions is also associated with disorders in comprehension and the identification of sounds related to human actions. Crucially, these symptoms are topographically specific, as patients with deficits in performing limb and bucco-facial actions are impaired in matching limb and mouth action-related sounds, respectively (Pazzaglia, Smania, et al., 2008b). Thus, it seems that motor production modulates action recognition, no matter whether it is mediated through visual, auditory or multimodal sensory inputs.

Studies on SCI support this notion. Although these patients often report that they walk in their dreams (Saurat et al., 2011), there is evidence indicating that paraplegic patients may suffer from a dramatic reduction in their motor imagery capacities (Alkadhi et al., 2005; Chen et al., 2016; Di Rienzo, Collet, et al., 2014a; Di Rienzo, Guillot, et al., 2014b; Hotz-Boendermaker et al., 2008; Scandola, Aglioti, Pozeg, et al., 2017b) and in the discrimination of biological motion (e.g., the direction of ambulation of a point-light walker; Arrighi et al., 2011), even if they are aware of their motor deficits (Manson et al., 2014). Again, these disorders in action representation may be topographically specific, involving actions that would be executed by the paralysed below-lesion body parts but not those performed by the above-lesion body parts (Pernigo et al., 2012; Scandola, Aglioti, et al., 2019a). Thus, impairment of motor simulation seems to be linked to failures in motor imagery. Interestingly, studies indicate that people with SCI fail in motor imagery only when they are asked to carry out the task by assuming a first-person perspective (i.e., internal, first person visual imagery and kinesthetic mental imagery), while they do not differ from controls when the task is executed assuming a purely visual, third-person perspective (i.e., external motor imagery; Scandola, Aglioti, Pozeg, et al., 2017b), a condition in which they declare that they use strategies based on memory. This result should be taken into account when devising rehabilitation training focused on motor imagery as disorders in this ability may be present and impact on the efficacy of the training. To date, results from motor imagery interventions on pain severity are conflicting, while a certain degree of functional improvement has been found when mental imagery is combined with physical practice (for review, see Opsommer et al., 2020; Opsommer & Korogod, 2017).

A specific deficit in learning implicit motor sequences was also observed in SCI individuals by Bloch and co-workers (2016), who tested healthy people and SCI paraplegic participants (with normal motor and sensory functions in their upper limbs) in a task where they were requested to press some buttons on a keyboard according to sequences indicated by cues shown on a computer screen. The order of the buttons was pre-arranged, and the same sequence was repeated for six blocks of stimuli. At the seventh block the sequence changed, and in the eighth block the first sequence was repeated. The RTs of the responses to the eighth block showed a learning effect related to the task in healthy but not in SCI participants. These results are interpreted by the authors as a deficit of SCI individuals in building a new motor expertise (Bloch et al., 2016).

Particularly interesting for the aim of this review is the contrasting, complementary data coming from investigations of SCI patients that provide evidence that the motor abilities that paraplegics develop after lesion-onset and during rehabilitation can lead to new action discrimination skills. This was demonstrated in the study by Pernigo et al. (2012) in which paraplegics who regularly practise sports became particularly good at the visual discrimination of actions performed by the upper parts of their body. In other words, there is a correspondence between the body parts used in sports (e.g., in this case, the arms and the upper part of the trunk) and the actions that the participants were better able to perceive. The data were subsequently replicated (Scandola, Aglioti, et al., 2019a) by means of a Progressive Temporal Occlusion paradigm (Abernethy, 1987). A group of paraplegics were exposed to two series of videos showing a person in a wheelchair or on rollerblades who was trying to climb on a step. The participants were asked to predict how the video would end choosing from three alternatives in which the person in the video: (i) carried out the action successfully; (ii) was not able to get onto the step or (iii) fell to the ground. The performance of the SCI participants was compared to that of two other groups. One control group was composed of physiotherapists with experience in the rehabilitation of people with SCI, but who were inexperienced with rollerblades. The experimental paradigm allowed the experimenters to exclude the possibility that mere visual expertise or knowledge of the kinematics involved in the use of a wheelchair influenced the performance of the SCI participants in the videos involving a wheelchair. The other control group consisted of experienced rollerblade skaters who were, however, inexperienced with wheelchairs. This permitted the experimenters to compare the performance of the SCI group in the videos showing a rollerblader with that of a group of experts. The hypothesis was that if abilities relating to action anticipation are modulated by the person’s sensorimotor experience, the paraplegic group would perform more accurately in the wheelchair videos and the group of skaters in the rollerblade videos. This was precisely what the main results of the study demonstrated (with the group of physiotherapists performing with average accuracy in both videos, Scandola, Aglioti, et al., 2019a).

Differences were also found in an affordance-related reachability judgment task (Sedda et al., 2018) in which only the control group overestimated the range. In addition, while the controls were faster at making judgments on reachability when the objects were in their peripersonal (vs. extrapersonal) space, the SCI patients did not seem to have this advantage for objects that were close. Importantly, this finding was related to the patients' ability to perform everyday tasks.

As a whole, these data indicate that de-afferentation and de-efferentation after spinal cord lesions impact body and action knowledge not only at the lower level of perception and execution, but also at the level of higher cognitive processes relating to representation, visual discrimination and mental imagery.

## How can the study of SCI contribute to the debate on the embodied cognition approach?

In the previous sections, we have shown that de-afferentation and de-efferentation due to SCI do not impact only somatosensory and motor functions but extend to higher-order body- and space-related cognitive functions. In doing so, a progressive approach was followed, moving from the more expected changes in body perception towards modifications at representational levels relating to knowledge of objects and space. Experimental data also show that, although a SCI results in a reduction in functional autonomy, the changes in the body-cognition relationship may also lead to the learning of new abilities which are very specific to the patient’s new post-lesional condition. It could be argued that these effects are not immediately obvious and in fact specific experimental procedures are required in order for them to be seen. Indeed, the experiments carried out in this area confirm what SCI patients have spontaneously reported on the subject of their daily experiences and the efforts they need to make in order to deal with their new condition (Conomy, 1973; Murphy, 1990; Papadimitriou, 2008; Scandola, Aglioti, Avesani, et al., 2017a).

Along the continuum of the different positions shown in Fig. 1, the results of tests with SCI patients support the ideas that cognition depends on the experience of having a body with sensorimotor capacities (Varela et al., 1991), and that cognition and representational processes are built on sensorimotor information (Barsalou, 2008; O’Regan & Noë, 2001). The notion that in SCI patients this knowledge is at least partly experience-dependent (Ostarek & Bottini, 2021) is supported not only by the loss of abilities but also (and probably in a stronger way) by data on the post-lesional acquisition of new abilities (e.g., the capacity to discriminate wheelchair actions). Nevertheless, when considering the nature of human experiences, one needs to take into consideration not only sensorimotor but also cognitive and affective experiences. Thus, the role of the body is difficult to isolate. It is worth noting, however, that changes in body, space and action representation after SCI can be ‘modality specific’ (i.e., representations change as a consequence of sensorimotor deficits, without modifications in visual or other sensory systems, or in higher cognitive or affective functions). Furthermore, these changes are topographically organised, namely they only regard the de-afferented and de-efferented body parts. These specificities make it possible to conclude that, at least for these functions, cognition is embodied.

De-afferentation and de-efferentation do not only change the perception of one’s own body, but also impact the mental representation of the bodily self and the relationship that an individual has with objects and the environment. With respect to the body, there is a loss of the equilibrium between top-down and bottom-up processes, with either the latter or the former prevailing depending on the circumstances. With reference to the environment, SCI changes the individual’s relationship with the surrounding space.

Unfortunately, to date, the results coming from behavioural studies have not yet been supported by neurophysiological and neuroanatomical studies with respect to the possibly specific changes in brain networks. However, neuroimaging studies show that the neuroplastic reorganisation after SCI extends beyond the sensorimotor systems, and also affects neural networks involved in cognitive functions (Curt et al., 2002; Gustin et al., 2010; Solstrand Dahlberg et al., 2018; Vastano et al., 2021). Furthermore, although the results are still preliminary and need confirmation, a deterioration in cognitive functions such as attention, memory and executive functions has also been reported following SCI (Chiaravalloti et al., 2020; Guadagni et al., 2019; Molina et al., 2018). Crucially, in the absence of brain damage, the only possible explanation (although speculative at the moment) for these data is that bodily changes, and in particular sensorimotor deprivation, can modify the central networks involved in these cognitive functions.

With a view to improving knowledge regarding the role of the body in cognition, it seems particularly important to investigate the role of sensory channels (e.g., sight and hearing) and interoceptive inputs coming from visceral organs (not affected by SCI) in the reorganisation of the body-cognition relationship in de-afferented/de-efferrented people. This research area promises to contribute substantially to the debate on embodied cognition theories.

## Conclusions and future research

Taken as a whole, the results from research in SCI indicate that sensorimotor functions have an important role in cognition since a body-brain disconnection modifies not only the individual’s mental representations of their body but also their knowledge of the environment around them. This may contribute towards a better understanding of the body-cognition relationship and further support the embodied approach to the study of cognition. Further studies are necessary to understand if these changes also involve symbolic functions such as language and social cognition. Moreover, future investigations should address the important issue of whether spared sensory systems, and in particular information coming from inside the body (i.e., interoception), may contribute towards the maintenance or rebuilding of the representation of one’s own body in the environment.
